# The Segregated Expression of Voltage-Gated Potassium and Sodium Channels in Neuronal Membranes: Functional Implications and Regulatory Mechanisms

**DOI:** 10.3389/fncel.2017.00115

**Published:** 2017-04-24

**Authors:** Maël Duménieu, Marie Oulé, Michael R. Kreutz, Jeffrey Lopez-Rojas

**Affiliations:** ^1^Research Group Neuroplasticity, Leibniz Institute for NeurobiologyMagdeburg, Germany; ^2^Leibniz Group “Dendritic Organelles and Synaptic Function”, University Medical Center Hamburg-Eppendorf, Center for Molecular Neurobiology (ZMNH)Hamburg, Germany

**Keywords:** voltage-gated sodium channels, voltage-gated potassium channels, polarized trafficking, compartmentalization, voltage-gated ion channels, axon initial segment, dendrites, node of Ranvier

## Abstract

Neurons are highly polarized cells with apparent functional and morphological differences between dendrites and axon. A critical determinant for the molecular and functional identity of axonal and dendritic segments is the restricted expression of voltage-gated ion channels (VGCs). Several studies show an uneven distribution of ion channels and their differential regulation within dendrites and axons, which is a prerequisite for an appropriate integration of synaptic inputs and the generation of adequate action potential (AP) firing patterns. This review article will focus on the signaling pathways leading to segmented expression of voltage-gated potassium and sodium ion channels at the neuronal plasma membrane and the regulatory mechanisms ensuring segregated functions. We will also discuss the relevance of proper ion channel targeting for neuronal physiology and how alterations in polarized distribution contribute to neuronal pathology.

## Introduction

The neuronal cytoarchitecture defines two main cellular compartments: dendrites that receive, integrate and propagate synaptic input (Häusser et al., 2000; Magee and Johnston, 2005; Stuart and Spruston, 2015) and the axon that eventually converts these processed inputs into variable patterns of action potential (AP), with fast and robust transmission to distant postsynaptic targets. Whereas some ion channels can be found all along the neuronal plasma membrane (Lim et al., 2000; Trimmer and Rhodes, 2004; Kirizs et al., 2014), others exhibit a more restricted expression pattern to either the axon or the somatodendritic region (Kerti et al., 2012). The functional properties of each cellular compartment and membraneous subcompartment critically depend on the type of voltage-gated ion channels (VGCs) inserted in the corresponding membrane (Lai and Jan, 2006; Beck and Yaari, 2008; Remy et al., 2010).

This review article summarizes current knowledge regarding potassium and sodium channels trafficking and surface expression and highlights the remaining questions regarding the mechanisms by which segregation of these ion channels at the plasma membrane is achieved. We focus on specific potassium and sodium VGCs: Kv1, Kv4.2 and Kv2.1 and Nav1.2, Nav1.6, respectively. These channels are critical in controlling neuronal intrinsic excitability and are among the best investigated VGCs (Vacher et al., 2008; Catterall, 2012; Vacher and Trimmer, 2012; Trimmer, 2015; Table 1). Principles underlying their polarized distribution and function in neurons are better understood and might be paradigmatic to understand the mechanisms controlling the subcellular distribution and function of other ion channels present in axons and dendrites.

**Table 1:** **Summary of localization, accessory subunits and physiological functions of voltage-gated potassium and sodium channels**.

Ion channels	Localization	Accessory proteins	Physiological functions
**Kv1.1, Kv1.2**	Distal AIS and juxtaparanode	Kvβ2 subunit	Regulation of AP threshold. Membrane repolarization. Spatially restrict propagation of excitation.
**Kv1.4**	Distal AIS, juxtaparanode and presynaptic sites	Kvβ2 subunit	Mediates a fast hyperpolarizing current. Associates with AIS- and nodal-Kv1.1/Kv1.2 heteromers, influencing their surface expression.
**Kv2.1**	Proximal dendrites, soma and AIS	AMIGO	Single channels: regulate high frequency firing. Clustered channels: non-conducting, but contribute to the excitation-gene transcription coupling.
**Kv4.2**	Spines, dendrites and soma	DPP6/DPPX and KChIPs	Dampens the propagation of depolarizing signals.
**Nav1.2**	AIS and nodes of Ranvier_(immature axon)_ Proximal AIS_(mature axon)_	β_1–4_ subunit	AP generation _(immature axon)_ AP back-propagation_(mature axon)_
**Nav1.6**	Dendrites, soma, distal AIS and nodes of Ranvier	β_1–4_ subunit	Generation of dendritic spikes. High efficiency axonal AP generation.

*For clarity, the table refers mainly to CA1 pyramidal cells*.

## Segregated Distribution of VGCs in Dendrites

### Kv4.2 and Nav1.6 Channels in Dendrites

The structural properties of dendrites, i.e., thickness and number of branches among others, define in part how they conduct and integrate synaptic signals (Segev and Rall, 1998; Stuart and Spruston, 2015). In addition, the expression pattern of different classes of VGCs is a key feature that determines the electrical properties of dendrites (Lai and Jan, 2006; Trimmer, 2015). Potassium channels are the most diverse family of ion channels and are present throughout the brain (Lai and Jan, 2006; Luján, 2010; Trimmer, 2015). These channels are composed of several pore-forming α subunits that interact with auxiliary subunits such as K^+^ channel Interacting Proteins (KChIPs), DPPX, Kvβ and AMIGO. The association of the α subunits with the auxiliary subunits changes the electrophysiological and biophysical properties of the channels (An et al., 2000; Bähring et al., 2001; Shibata et al., 2003; Peltola et al., 2011) and can also affect their expression level and distribution pattern (An et al., 2000; Shibata et al., 2003). At the functional level, potassium channels in dendrites are key regulators of dendritic excitability as they strongly filter and shape electrical signals traveling between synapses and the soma and all the way back from soma to synapses (Watanabe et al., 2002; Takigawa and Alzheimer, 2003; van Welie et al., 2004; Misonou et al., 2005b; Chen and Johnston, 2006; Kim and Hoffman, 2008).

Regarding sodium channels, to date nine Nav channels α subunit isoforms have been identified (Nav1.1–1.9), each channel being composed of one α subunit with four domains that form the pore of the channel. Nav channels also interact with auxiliary β subunits (β_1–4_) that regulate their trafficking or biophysical properties (Patino and Isom, 2010; O’Malley and Isom, 2015). Whereas the expression pattern and physiological function of Nav channels are relatively clear in the axon (see the corresponding section for more details) their localization in the somatodendritic compartment is not well investigated. Nonetheless, upon synchronous stimulation dendritic Nav channels can generate local dendritic spikes in pyramidal cells (Golding and Spruston, 1998; Spruston, 2008; Sun et al., 2014; Kim et al., 2015), a regenerative mechanism that has been shown to facilitate synaptic plasticity in CA1 (Kim et al., 2015) and CA2 (Sun et al., 2014) pyramidal neurons.

#### The Proximodistal Dendritic Gradient of Kv4.2 and Nav1.6 Channels Influence Distance-Dependent Dendritic Integration of Synaptic Signals

**Figure 1:**
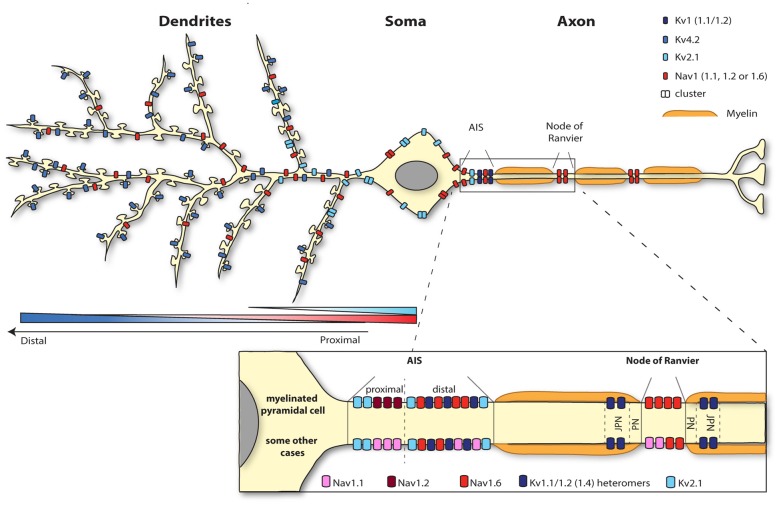
**Segregation of the neuronal surface expression of potassium and sodium channels**. Kv4.2 channels are expressed all along the dendrites and spines of pyramidal cells according to an increasing proximodistal gradient. Kv2.1 channel expression is restricted to the soma and the very proximal dendrites. Sodium channels are highly concentrated at the level of the axon initial segment (AIS; Nav1.1, 1.2 and 1.6) and nodes of Ranvier (1.2 and 1.6). Whereas myelinated pyramidal cells express Nav1.2 and Nav1.6 at the level of the AIS, Nav1.1 as well as Nav 1.2 and 1.6 are present at the AIS region of retinal bipolar cells, retinal ganglion cells, parvalbumine-positive interneurons (neocortex and hippocampus), Purkinje cells and spinal cord motoneurons for instance. Nav1.6 is also expressed in the somatodendritic compartment (but not spines) of CA1 pyramidal cells following a decreasing proximodistal gradient.

Upon membrane depolarization, the opening of the voltage-gated Kv4.2 channel generates the A-type current (Chen et al., 2006), dampening dendritic electrical signals (Hoffman et al., 1997). The importance of this channel in dendritic integration is further illustrated by the key role of its downregulation in the induction and expression of LTP in CA1 pyramidal cells (Frick et al., 2004; Chen et al., 2006), as well as its role in memory formation (Lugo et al., 2012; Truchet et al., 2012; Vernon et al., 2016). Kv4.2 is found on somatic and dendritic membranes, as well as in spines of CA1 pyramidal cells (Jerng et al., 2004; Rhodes et al., 2004; Kerti et al., 2012; Figure 1). Although immunogold staining have shown slight differences in the density of Kv4.2 labeling between proximal and distal dendrites of CA1 pyramidal cells (Kerti et al., 2012), the actual A-type current is much larger at the very distal dendrites compared to the proximal ones (Hoffman et al., 1997). The discrepancy between the Kv4.2 expression pattern and dendritic A-type current amplitude suggests different regulation of the channel depending on its location along the proximodistal dendritic axis.

Concurrently, upon membrane depolarization the opening of sodium channels further depolarizes the membrane contributing to an enhanced excitability. From all sodium channels, Nav1.6 is the main subtype identified in the dendritic membrane of CA1 pyramidal cells, suggesting that this channel is responsible for the generation of the dendritic sodium spikes in this cell type. Nav1.6 is expressed throughout the neuron, from the nodes of Ranvier to dendrites (Caldwell et al., 2000; Krzemien et al., 2000; Figure 1). Since the intensity of labeling is much weaker on dendrites (around 40 times less) than in the axon (Lorincz and Nusser, 2010), Nav1.6 was not always detected in immunolabeling studies (Lorincz and Nusser, 2008; Hu et al., 2009). A recent study using electron microscopy has revealed that Nav1.6 channel is indeed present in the somatodendritic compartment, but excluded from spine synapses, of CA1 pyramidal cells with a decreasing proximodistal gradient of expression (Lorincz and Nusser, 2010; Figure 1).

Thus, Kv4.2 and Nav1.6 channels exhibit opposing proximodistal expression patterns and the prominent expression of Kv4.2 together with the weak expression of Nav1.6 in distal dendrites reduces the likelihood of distal synaptic signals to reach the soma. In contrast, the large density of Nav1.6 channels and the reduced expression of Kv4.2 channel in proximal dendrites make the transmission of synaptic stimuli impinging on this area much more reliable. Intuitively, dampening the transmission of distal signals over proximal ones seems not to be meaningful for dendritic integration. The physiological relevance for dendritic integration of setting such ion channel gradients still remain to be fully understood. Nevertheless, downregulation of Kv4.2 channel is crucial for local dendritic plasticity of CA1 pyramidal cells, and specifically for the modulation of dendritic sodium spikes, potentially mediated by Nav1.6 channel (Losonczy and Magee, 2006; Losonczy et al., 2008; Weber et al., 2016). Hence, concomitant regulation of the surface expression or activity of both channels appears to be a major process for shaping dendritic integration.

#### Molecular Mechanisms Setting Dendritic Proximodistal Expression Gradients

##### Segregated targeting of ion channels to the somatodendritic compartment

**Figure 2:**
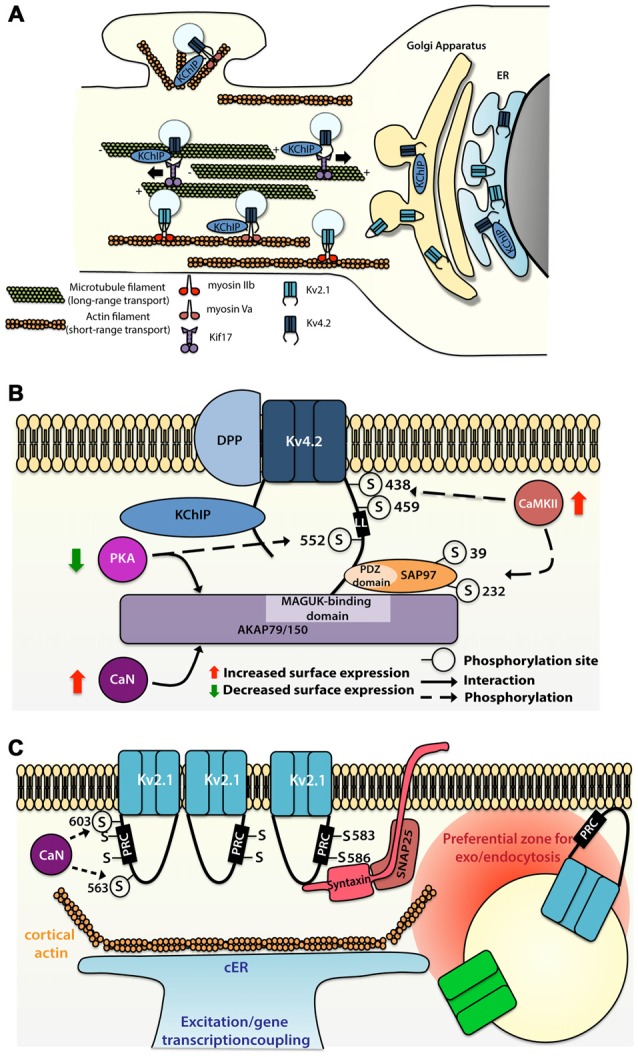
**Molecular mechanisms allowing segregated expression of the dendritic Kv4.2 and Kv2.1 channels. (A)** Sorting and trafficking of Kv4.2 and Kv2.1 channels. Association of Kv4.2 channel with auxiliary protein K^+^ channel Interacting Protein (KChIP) targets the channel to the cell surface. Kif17 ensures microtubule-based transport whereas myosin Va is used for actin-based transport of Kv4.2 channel on proximal dendrites and spines. Myosin IIb transports Kv2.1 to the somatodendritic compartment. **(B)** Intrinsic motifs, posttranslational regulations and binding partners regulating Kv4.2 surface expression. **(C)** Intrinsic motifs, posttranslational regulations and binding partners regulating Kv2.1 cluster formation and function.

Two main classes of auxiliary subunits are known to interact with the Kv4.2 channel: the KChIP (Shibata et al., 2003; Rhodes et al., 2004) which most likely interact with the intracellular N-terminus of the channel (for review Jerng et al., 2004) and the Dipeptidyl Peptidase-like Protein (DPP) subunit (Nadal et al., 2003; Kim et al., 2008) whose interaction is proposed to be mediated by the transmembrane domain of the DPP protein and the voltage sensor domain of the potassium channel (Ren et al., 2005; Zagha et al., 2005). Kv4.2 co-localizes with KChIP2 in apical and basal dendrites of hippocampal and cortical pyramidal cells (Rhodes et al., 2004) and KChIP1-3 associate with the Kv4.2 channel to promote its surface expression by facilitating the release of the channel from the endoplasmic reticulum (ER; Shibata et al., 2003). This mechanism might involve the masking of the cytoplasmic ER retention signal located in the N-terminal domain of the α subunit, raising the idea that the N-terminus of the channel is necessary for both binding to KChIPs and ER retention (Shibata et al., 2003; Figure 2A). Interestingly, due to the unique presence of a K-channel inactivation suppressor (KIS) domain on the KChIP4a (Holmqvist et al., 2002), this auxiliary subunit does not promote the trafficking of Kv4.2 channel outside of the ER (Shibata et al., 2003). But the coassembly of KChIP4a with Kv4.2 and other KChIP subunits allows the complex to be expressed at the surface level (Shibata et al., 2003). Indeed in COS7 cells phosphorylation of a specific serine residue (S552) of the Kv4.2 channel by PKA leads to an increased surface expression of the channel in complex with KChIP4a (Lin et al., 2011). Thus, it seems that surface expression of Kv4.2 channel can be finely tuned depending on the stoichiometry of the coassembly of Kv4.2 channel with KChIP subunits, as well as the phosphorylation status of the channel. The DPPX auxiliary subunit, also called DPP6, a member of the DPP protein family, is expressed in the same population of neurons than Kv4.2 channel and its expression is also restricted to the somatodendritic compartment. In CHO cells, the coexpression of Kv4.2 channel with DPPX targets the channel at the cell surface, whereas Kv4.2 channel expressed alone is sequestered in the perinuclear ER (Nadal et al., 2003). DPPX presents homology with the CD26 protein (Nadal et al., 2003), known for its role in cell adhesion and interaction with the extracellular matrix (ECM; Hildebrandt et al., 2000). The DPPX homologous extracellular cysteine-rich domain is thought to be important for the extracellular regulation of the trafficking of Kv4.2 channel in complex with DPPX (Nadal et al., 2003). Even though Kv4.2 auxiliary subunits are critical for the targeting of the channel to the somatodendritic compartment, how these proteins influence the segregated expression of the channel still remains elusive.

Microtubule-based dendritic transport of Kv4.2 is supported by the kinesin Kif17 in cortical cells cultures, most likely through interaction with the C-terminus (but not the Kv4.2 dileucine motif; Chu et al., 2006), whereas association of Kv4.2 with myosin Va, an actin-based motor, restricts the expression of the channel to the somatodendritic region (Lewis et al., 2009; Figure 2A). Comparison of the trafficking mechanism of Kv4.2 and Kv2.1 channels have shown that channels are sorted at the Golgi apparatus into different vesicle pools, each pool being transported in a compartment-specific manner (Jensen et al., 2014). Jensen et al. (2014) have demonstrated in hippocampal primary neurons that mutation of the C-terminal domain of the channels (Kv4.2LL/AV and Kv2.1S586A) causes their mislocalization, with expression of the Kv4.2 channel at the somatodendritic compartment as well as in the axon, and expression of the Kv2.1 all along the dendritic plasma membrane. Interestingly, Jensen et al. (2014) also reported that disruption of actin polymerization using latrunculin A alters the motility of Kv2.1-contaning, but not Kv4.2-containing vesicles. However blocking actin polymerization with cytochalasin D led to a diffuse targeting of the Kv4.2 channel in a study by Lewis et al. (2009). These discrepant observations raise questions regarding the role of actin in the transport of Kv4.2-containing vesicles. Nevertheless, one hypothesis could be that actin-based transport is used for short distance transport to the proximal dendrites or from dendritic shaft to the spine, whereas Kv4.2 channels expressed distally are trafficked based on long distance microtubule-based transport (Figure 2A).

Rivera et al. (2003) identified a dileucine-containing motif in the C-terminus of Kv4.2 as sufficient and necessary for the dendritic expression of the channel (Figure 2B). Interestingly, elimination of the dileucine-containing motif causes the targeting of the channel predominantly at cell body, and to a lesser extent at the proximal part of both dendrites and axon, thereby suggesting cell body “default-targeting” of the channel (Rivera et al., 2003).

Several members of the Kv family have been shown to interact with proteins located at the postsynaptic density, most notably the membrane-associated guanylate kinases (MAGUKs; Gardoni et al., 2007; Lin et al., 2011; Fourie et al., 2014). In CA1 pyramidal cells, the Kv4.2 channel also associates with the A-kinase-anchoring protein (AKAP) complex, providing local coupling with kinase and phosphatase signaling. AKAP79/150, a member of the AKAP family, interacts with Kv4.2 channel through its MAGUK-binding site, but this interaction does not require the interaction of Kv4.2 channels with the MAGUK PDZ domains (Lin et al., 2011). Whereas association of PKA with AKAP79/150 decreases the surface expression of the Kv4.2 channel, the binding of calcineurin with the AKAP complex potentiates its surface expression (Lin et al., 2011). In parallel, Kv4.2 channel also interacts with other synaptic proteins, such as SAP97, through the C-terminus of the channel and the PDZ domain of the SAP97 protein (Gardoni et al., 2007). Findings from this study indicate that CaMKII-dependent Ser39 and Ser232 phosphorylation of the SAP97 protein increases the expression of the Kv4.2 channel at dendritic spines (Gardoni et al., 2007), suggesting that CaMKII-dependent SAP97 phosphorylation is important for synaptic trafficking of Kv4.2 channel (Figure 2B).

The Kv4.2 channel contains various residues that can be phosphorylated by diverse kinases such as PKA, PKC, CaMKII and ERK (Adams et al., 2000; Anderson et al., 2000; Varga et al., 2004; Schrader et al., 2009). While CaMKII-mediated phosphorylation of Ser438 and/or Ser459 enhances the surface expression of Kv4.2 channel (Varga et al., 2004), PKA-mediated phosphorylation of the Ser552 decreases the surface expression of the channel (Hammond et al., 2008). A study suggests that phosphorylation could be used as well as a discrete tag for targeting. Indeed, the Kv4.2 channel exhibits different phosphorylated sites (ERK sites, N-terminal PKA sites or C-terminal PKA sites) depending on their localization along the dendritic tree of different cell types (Varga et al., 2000).

Contrary to the axonal compartment, in dendrites the expression pattern, trafficking and insertion mechanisms of Nav channels remain largely elusive. Combination of fluorescence techniques with high-density single-particle tracking has revealed that Nav1.6 channels are organized as small clusters (~230 nm) formed of 2–20 Nav1.6 channels at the surface of the soma (Akin et al., 2015; Figure 1). Strikingly, the removal of the ankyrin-G (AnkG)-binding motif of the Nav1.6 channel, crucial for its localization at the axon initial segment (AIS) region (Gasser et al., 2012), does not alter the formation of the nanoclusters observed at the surface of the soma (Akin et al., 2016). Cluster formation was also actin-independent and did not occur at the vicinity of the Kv2.1-mediated membrane trafficking hubs that are observed at the surface of the neuronal membrane (Akin et al., 2016). The AnkG-independent surface expression of the Nav1.6 channel in the soma of CA1 pyramidal cells suggest that compartment-specific binding partners could direct the expression of Nav1.6 channel to either the somatodendritic region or the axonal one.

It should be noted that Nav1.1 has been as well identified in the somatodendritic compartment of CA1 pyramidal cells (Westenbroek et al., 1989; Gong et al., 1999; for review, see Trimmer and Rhodes, 2004; Vacher et al., 2008). However, a more recent study from Lorincz and Nusser (2010) did not find Nav1.1 subunit expression in CA1 pyramidal cells, but only on axonal processes and AIS of GABAergic interneurons.

##### Establishment of dendritic proximodistal gradient and activity-dependent regulation of ion channels surface expression

In addition to its role in the surface targeting of the Kv4.2 channel, the auxiliary subunit DPP6/DPPX is thought to be important for the establishment of the proximodistal gradient of expression of Kv4.2. Knock-out of this auxiliary subunit leads to a reduced A-type current in the distal dendrites (Sun et al., 2011). However, the molecular mechanism through which DPP6 contributes to set such a gradient is unknown.

In cultured hippocampal neurons Kv4.2 channels can be removed from spines, where they are supposed to be enriched (Kim et al., 2005), through clathrin-mediated internalization triggered by NMDAR activation and increase in calcium-influx into the cell (Kim et al., 2007). Using a FRAP assay on CA1 pyramidal cells, Nestor and Hoffman (2012) have demonstrated that Kv4.2 mobility is positively regulated by AMPAR-dependent induction of PKA-mediated phosphorylation of Ser552 targeting the channel for clathrin-mediated internalization only at the distal dendrites (Figure 2B). Dynamic regulation of Kv4.2 channel surface expression fits with previous studies showing that activity-induced downregulation of the channel facilitates dendritic integration (Losonczy and Magee, 2006; Weber et al., 2016), helping synaptic signals to overcome the proximodistal gradient of Kv4.2.

### The Somatodendritic Voltage-Gated Kv2.1 Channel forms Segregated Membrane Clusters that Participate to Novel Ion Channels Insertion and Retrieval

The Kv2.1 channel, a potassium channel that mediates the majority of the delayed-rectifier K^+^ currents (Murakoshi and Trimmer, 1999), regulates membrane excitability during high frequency firing (Du et al., 2000; Misonou et al., 2005b). It is abundantly expressed throughout the brain and is particularly prominent in the hippocampus (Murakoshi and Trimmer, 1999; Antonucci et al., 2001). Several immunohistochemical studies have shown that Kv2.1 is expressed at similar levels in the AIS, soma and very proximal part of the dendrites (Lim et al., 2000; Trimmer and Rhodes, 2004; Kirizs et al., 2014; Figure 1). Kv2.1-containing vesicles traffic on actin filaments through an association with myosin IIb (Jensen et al., 2014; Figure 2A). Interestingly, it has been observed that mutation of S586 of the Kv2.1, which is important for its expression at the somatodendritic membrane, does not alter surface expression at the AIS (Jensen et al., 2014), suggesting somatodendritic and axonal compartment-specific mechanisms, at least partially independent from each other, allowing the sorting of the channel to one of those regions.

Compared to other Kv channels, the Kv2.1 channel has the peculiarity to associate in large clusters (1–2 μm) at the membrane surface (Trimmer, 1991; Scannevin et al., 1996; O’Connell et al., 2006; Tamkun et al., 2007), whereas other channels show a more diffuse localization (Tamkun et al., 2007; Figure 2C). Given its pivotal role in the regulation of high frequency firing and its ubiquitous brain expression, cellular and molecular mechanisms regulating the expression and targeting of Kv2.1 channel have been extensively studied. A Proximal Restriction and Clustering signal (PRC signal) has been suggested to mediate the surface expression pattern of the Kv2.1 channel (Lim et al., 2000; Figure 2C). This uncommon signal in the cytoplasmic domain, which is rich in serine and threonine residues (7/26 positions), does not contain tyrosine or di-leucine motifs required for endosomal sorting and is supposed to exclusively ensure the clustering of the Kv2.1 channel at the surface membrane. The precise mechanism by which the PRC signal targets and sets the clustering of the Kv2.1 channel is not yet known. In addition it was shown that the interaction between both N-and C-termini of the Kv2.1 channel is necessary for efficient targeting of the channel at the membrane surface and this interaction is mediated by regions in the N- and C-termini that are normally involved in the interaction with auxiliary subunits in other classes of potassium channels (Mohapatra et al., 2008).

Surprisingly, Tamkun et al. (2007) showed that Kv2.1 channels have comparable lateral mobility at the plasma membrane irrespective of whether those channels were part or not of the clusters. This observation suggests that no static anchoring with classical scaffolding proteins occurs arguing in favor of a corral-forming fence restraining the diffusion of the channel. In some cases, Kv2.1 channels outside the clusters could readily diffuse within the cluster where they could be trapped and confined for up to an hour (Tamkun et al., 2007). The authors proposed a model in which Kv2.1 channels interact through their C-termini with accessory proteins located beneath the membrane, potentially in a phosphorylated-dependent manner, defining a cluster located within cortical actin wells.

While non-clustered Kv2.1 channel are responsible for the high-threshold delayed-rectifier current, clustered Kv2.1 channels are non-conducting but are instead able to sense membrane potential linking membrane potential changes to intracellular signaling cascades (O’Connell et al., 2010; Fox et al., 2013b). An interesting aspect of the Kv2.1 channel localization is its close association with subsurface cisternae (Fox et al., 2013a, 2015)—intracellular ER-derived membranes—that buffer and store intracellular calcium necessary for propagation of signaling cascades important for the regulation of neuronal trafficking (Figure 2C). Subsurface cisternae are called cortical endoplasmic reticulum (cER) and are suggested to form a hub that supports the trafficking of plasma membrane proteins (Fox et al., 2013a). In HEK cells Kv2.1 channel initiates the formation of this plasma membrane-ER junction (Fox et al., 2015) thanks to its remodeling, while Kv2.1 declustering upon glutamate stimulation led to retraction of cER away from plasma membrane, suggesting that the association of Kv2.1 channel with subsurface cisternae could couple electrical membranous events with intracellular calcium homeostasis. Moreover, preventing channel clustering by mutating two serine residues (S583 and S586), located within the C-terminal PRC sequence of Kv2.1 channel, also blocks cER remodeling (Figure 2C). Deutsch et al. (2012) demonstrated that Kv2.1-contaning vesicles actually tether and deliver cargo at the vicinity of the channel clusters in both HEK cells and cultured hippocampal neurons. Furthermore, quantum dot analysis showed that delivery and recycling of Kv2.1 channel occurs in a perimeter of 0.5 μm away from the cluster fence. Strikingly, they observed that Kv2.1 channel clusters are also used as a trafficking platform for insertion and retrieval of the non-clustering Kv1.4 channels (Deutsch et al., 2012) and later Cav1.2 channel has also been shown to be located in close proximity of Kv2.1 channel (Fox et al., 2015). Altogether these observations led to the speculation that these clusters are preferential locations for the exo- and endocytosis of different ion channels (Figure 2C), an important process that could participate to the segregation of ion channels membrane expression. The potential role of Kv2.1 channel in non-conducting phenomena is also supported by other observations. Some studies have focused on the role of Kv2.1 channel in vesicle-plasma membrane fusion (Feinshreiber et al., 2009, 2010). Of interest, Kv2.1 channel can interact with syntaxin and SNAP-25 (Figure 2C), two SNARE family proteins, which are known for their prominent role in vesicle fusion (Ramakrishnan et al., 2012; Südhof, 2013; Vardjan et al., 2013), supporting the idea that Kv2.1 platforms promote the insertion and recycling of other membrane proteins. Along these lines, the lack of proper Kv2.1 channel cluster formation might lead to alteration of local plasma membrane identity and the associated downstream signaling cascade. Thus, disruption of Kv2.1 channel clustering might induce mislocalization of other proteins and thereby dysfunction of dendritic signaling.

## Compartmentalized Distribution of VGCs in Axons

### Nav Channels

#### Distribution and Function of Axonal Nav Ion Channels

Although Nav1.1 is mostly somatodendritic in principal cells, it is the dominant isoform in AIS of various GABAergic interneurons (Ogiwara et al., 2007; Lorincz and Nusser, 2008; Catterall et al., 2010; Tian et al., 2014) and is localized at the AIS and nodes of retinal cells (Van Wart et al., 2007; Puthussery et al., 2013) and motor neurons (Duflocq et al., 2008, 2011). In these cell types, Nav1.1 channel localization in the AIS region was mainly restricted to a narrow proximal domain in contact with the soma (Van Wart et al., 2007; Duflocq et al., 2008, 2011; Lorincz and Nusser, 2008), suggesting that this channel might regulate voltage propagation between the AIS and the somatodendritic compartment. The exact function of Nav1 channels expressed at the AIS and nodes of Ranvier in these cell types is still unclear but its dysfunction leads to variable disorders ranging from epilepsy (Wimmer et al., 2010) to autism and paralysis (Arancibia-Carcamo and Attwell, 2014). Nav1.3 is mostly absent in mature neurons but is present in the axon of dorsal root ganglion (DRGs) neurons where its expression is upregulated following injury and has been implicated in pain disorders (Lindia et al., 2005; Cummins et al., 2007). Expression of Nav1.4 has been so far only shown in skeletal muscles. Nav1.5 is mostly found in the heart, even though it has been reported to be punctually expressed in the brain (Wu et al., 2002) but not much is known about its neuronal function. Nav1.7, Nav1.8 and Nav1.9 are differentially expressed in various subtypes of DRGs sensory neurons, possibly underlying their respective functions (Vacher et al., 2008). They have been less studied than central nervous system (CNS) neurons isoforms but their role in pain signaling make them target of further research (Cummins et al., 2007; Bao, 2015).

Nav 1.2 and Nav1.6 are the most prominent isoforms expressed in axons of neuronal principal cells. Outside of the AIS and nodes of Ranvier, Nav1.2 seems rather uniformly localized along unmyelinated fascicles of both myelinated and unmyelinated axons in mature neurons (Caldwell et al., 2000; Boiko et al., 2001, 2003; Van Wart et al., 2007). In neuronal development it is also transiently expressed at the AIS and in nodes of Ranvier. In mature neurons it is replaced by Nav1.6 (Boiko et al., 2001, 2003; Ratcliffe et al., 2001; Rios et al., 2003). In mature cortical pyramidal neurons, Nav1.2 is maintained at the proximal AIS, segregated from Nav1.6 and it has been suggested to control back-propagation of APs to the soma (Dulla and Huguenard, 2009; Hu et al., 2009). The function of Nav1.2 in non-myelinated axon is unclear but it could in principle allow active propagation of spikes, supporting micro-saltatory or saltatory-like conduction of APs (Johnston et al., 1996; Caldwell et al., 2000; Zeng and Tang, 2009; Neishabouri and Faisal, 2014; Freeman et al., 2016; but see Black et al., 2002).

Nav1.6 channels are the main component of AIS and nodes of Ranvier in most CNS neurons (Boiko et al., 2001, 2003; Ratcliffe et al., 2001; Rios et al., 2003) and they have been shown to be the main controllers of spike generation (Hu et al., 2009). This is due to their hyperpolarized voltage-dependance, kinetic properties and increased persistent current as compared to Nav1.2, making it a more excitable isoform (Burbidge et al., 2002; Rush et al., 2005; Kole and Stuart, 2008; Hu et al., 2009). Nav1.6 shows similar functional properties as Nav1.2 when their respective α subunits are expressed in TsA-201 cells (Chen et al., 2008), suggesting that regulation of Nav1.6, possibly by β subunits, is required for their specific function. Nav1.6 is expressed in a gradually increasing concentration along the AIS and is predominantly enriched in the distal AIS (Inda et al., 2006; Kole et al., 2007; Van Wart et al., 2007; Lorincz and Nusser, 2008; Hu et al., 2009), which might be important to isolate the axon from the somatic capacitance and enhance sharpness of APs generation (Brette, 2013). It is tempting to speculate that similar to their role at the AIS, Nav1.6 control APs regeneration at nodes of Ranvier although this has not yet been directly demonstrated (Arancibia-Carcamo and Attwell, 2014).

#### Targeting Nav Channels to the Axon

**Figure 3:**
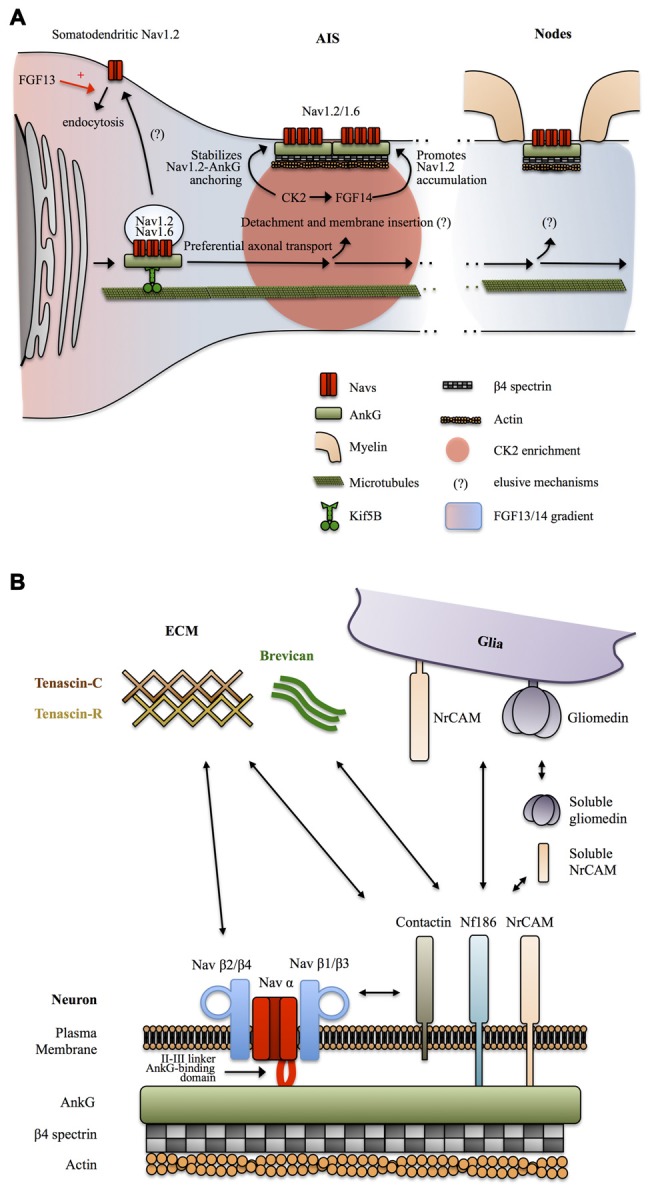
**Mechanisms targeting Nav channels to the axon. (A)** Preferential axonal transport and membrane insertion. Nav1.2, and presumably Nav1.6, are preferentially transported to the axonal domain by Kif5B. This is mediated by ankyrin-G (AnkG) which acts as a cargo adaptor. Casein kinase 2 (CK2) concentrates at the AIS following Nav channels enrichment and stabilizes the anchoring of Nav1.2 by phosphorylating their AnkG-binding domain. A somatodendritic-axonal gradient of fibroblast growth factor 13 (FGF13) and FGF14 participates in defining a polarized distribution of Nav1.2. FGF13 increases the endocytosis of Nav1.2 whereas FGF14 promotes their accumulation at the level of the AIS in a CK2-dependent manner. **(B)** Anchoring partners of Nav channels. β auxiliary subunits support Nav subcellular localization and surface expression stability by mediating multiple interactions with neuronal and glial transmembrane proteins but also components of the extracellular matrix (ECM).

Nav channels are clustered at the level of the AIS and nodes of Ranvier by tethering to AnkG (Bréchet et al., 2008; Brachet et al., 2010) which is linked to the cytoskeleton by direct interaction with the actin-binding protein β IV spectrin (Ratcliffe et al., 2001; Figures 3A,B). An AnkG binding domain has been first identified in the II-III cytoplasmic loop of the C-terminal region of the Nav1.2 α subunit (Garrido et al., 2003; Fache et al., 2004). This binding domain is conserved among Nav isoforms (Lemaillet et al., 2003) and has been shown to be sufficient to target Nav1.6 to AIS and nodes (Gasser et al., 2012). In addition, the Nav1.2 II-III linker contains an endocytosis signal that promotes clearance of Nav1.2 from the somatodendritic domains (Garrido et al., 2003; Fache et al., 2004; Figure 3A) but such signal has not yet been identified for Nav1.6 subunits (Garrido et al., 2001; Lai and Jan, 2006; Akin et al., 2015; Liu et al., 2015). This difference might be explained by the fact that Nav1.6 is expressed later in development and relies on more specific targeting mechanisms than Nav1.2.

How Nav channels get transported into the axon was unclear until a recent study suggests that AnkG and Nav1.2 associate early during biosynthesis and that AnkG acts as a cargo adaptor to mediate KiF5B/kinesin-1 based axonal transport of Nav1.2 in 7 DIV hippocampal neurons (Barry et al., 2014; Figure 3A). It was not directly assessed in this study if Nav1.6 is transported following the same AnkG-KiF5B interaction but published data support this idea. Nav1.6 is targeted to the AIS thanks to the conserved AnkG-binding domain of Navs (Gasser et al., 2012). In addition, Nav1.6 AIS targeting depends on vesicular trafficking (Akin et al., 2015). Finally axonal transport of Nav1.8 in DRGs neurons of the peripheral nervous system is also dependent on KiF5B (Su et al., 2013). The role of AnkG as a cargo adaptator and the contribution of this KiF5B-based vesicular transport to the maintenance of Nav in mature neurons was not directly assessed, but this appears likely since KiF5B maintains an axonal transport activity in mature neurons (Xu et al., 2010; Barry et al., 2014) and because AnkG is necessary for the maintenance of the AIS and Nav density (Hedstrom et al., 2008). Altogether, these studies suggest that early Nav-AnkG association in the ER and further cargo transport mediated by axonal-specific motors is an important determinant of Nav polarized expression (Figure 3A).

How the Nav-AnkG complexes get disattached from KiF5B and inserted in the plasma membrane at specific locations remains unknown (Jones and Svitkina, 2016). The interaction of Navs with AnkG is promoted by phosphorylation of their AnkG-binding domain by casein kinase 2 (CK2) which is enriched at the level of the AIS and nodes of Ranvier (Bréchet et al., 2008; Brachet et al., 2010). CK2 binds to the II-III intracellular loop of Navs. It was suggested that the enrichment of CK2 at the level of the AIS is actually a consequence of the dense concentration of Navs in this region (Hien et al., 2014). Non-canonical fibroblast growth factors (also called fibroblast growth factor homologous factors or intracellular fibroblast growth factor) FGF13 and FGF14 have recently been shown to be differentially expressed in hippocampal neurons in cultures and to be able to interact with the C-terminal domain of Nav1.2 channels. FGF13 promotes somatodendritic Nav1.2 channel endocytosis whereas FGF14 leads to their accumulation at the AIS (Pablo et al., 2016; Figure 3A). CK2 activity is required for the interaction between FGF14 and Nav1.2/Nav1.6 channels and also modulates Nav1.6 currents (Hsu et al., 2016). Interestingly, FGF14 is a target of Glycogen Synthase Kinase 3 (GSK3), an enzyme critically involved in neuronal polarity (Gärtner et al., 2006; Shavkunov et al., 2013). Altogether, these results indicate that Nav appearance at the AIS and in the somatodendritic domain is followed by post-translational mechanisms that stabilize Nav-AnkG complexes, maturate Nav kinetics and promote elimination of somatodendritic Navs, leading to further refinement of the polarized Navs distribution.

Sodium channels are also composed of transmembrane β subunits that associate with α subunits by covalent or non-covalent bonds and regulate surface expression and kinetics of Nav α subunits (Namadurai et al., 2015). Four β subunits β1, β2, β3 and β4 have been identified, with further complexity added by alternative splicing of β1 (Namadurai et al., 2015). These β subunits are structurally very close to the family of Cell Adhesions Molecules (CAMs; Srinivasan et al., 1998; Catterall, 2000; Chopra et al., 2007) and thus can link Nav channels with the extracellular space and mediate interactions with components of the ECM and with glia membrane proteins. β2 subunits are structurally close to the neurally expressed cell-adhesion protein contactin and can, like contactin, interact with tenascin C and R, proteins of the ECM, and promote surface expression of Nav1.2 (Srinivasan et al., 1998). Contactin has also been reported to directly bind Nav β1 and to co-localize with it at nodes in the CNS, during development, but also in the adult brain (Kazarinova-Noyes et al., 2001). The exact subcellular distribution and functions of tenascin C and R at the level of axonal domains are unknown. Tenascin C seems to be preferentially expressed during development, whereas tenascin R is also expressed during adulthood, both in an overall uniform extracellular manner (Gaal et al., 2015; Giblin and Midwood, 2015). One possibility is that tenascin C and R promote Nav channels, especially Nav1.2, surface expression during development and later in mature neurons define the uniform distribution of Nav1.2 in unmyelinated axons and unmyelinated tracks of myelinated axons. Overall, the composition of the ECM and its functional relevance is a relatively novel focus of research and definitive conclusions are still lacking. Clustering of Nav channels at mature AIS and nodes of Ranvier and perhaps as well in unmyelinated axons is most likely mediated by other CAMs. Indeed, Nav β1 and β3 subunits associate with NF186 (Ratcliffe et al., 2001), and NrCAM (McEwen and Isom, 2004) two CAMs expressed by neurons at the level of the AIS and nodes of Ranvier and possessing an AnkG-binding intracellular domain (Hedstrom et al., 2007). Neuronal NrCAM and NF186 in turn interact with glia-expressed NrCAM and gliomedin (Feinberg et al., 2010). In addition NrCAM and gliomedin can be secreted in a cleaved soluble form by glia (Feinberg et al., 2010). Complex heterophilic and homophilic interactions of both NrCAM (Mauro et al., 1992) and NF186 (Liu et al., 2011) make the exact interaction mechanisms difficult to address, but it appears that multiple interactions between glia and neuronal cell adhesion molecules regulate the clustering of Nav channels at the level of the AIS and nodes of Ranvier trough binding of their β1 and β3 subunits with NF186 and NrCAM and further coupling to AnkG and βIV spectrin-associated actin cytoskeleton (Feinberg et al., 2010). In addition, NF186 interacts with ECM proteins enriched at the level of the AIS and nodes of Ranvier such as the microglia and astrocytes secreted protein brevican (Hedstrom et al., 2007; Figure 3B).

### Kv Channels

**Figure 4:**
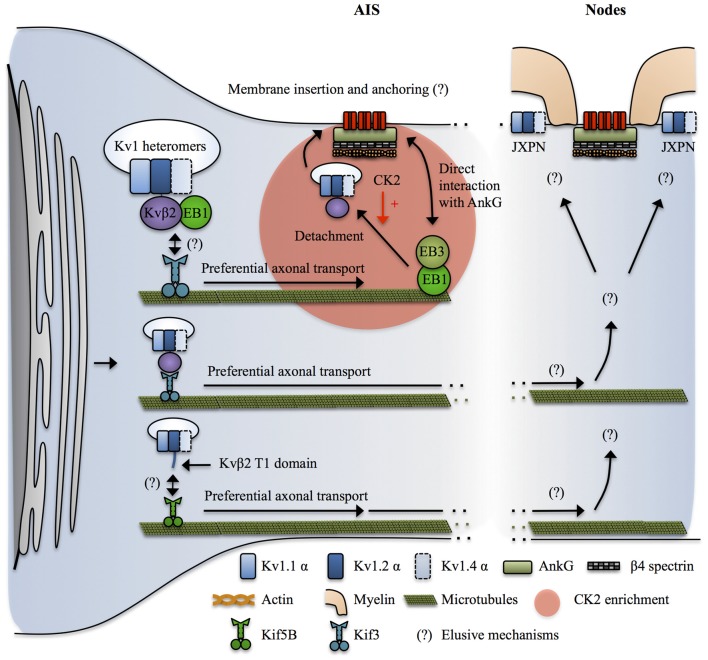
**Mechanisms targeting Kv1 channels to the axon**. Kvβ2 mediates the axonal targeting of Kv1.2 and Kv1.2-containing heteromers by acting as a cargo adaptor, or a binding partner for an undetermined cargo adaptator for Kif3. Kif3 preferentially transports the Kv1.2/Kvβ2-containing complexes to the axon by a mechanism that requires EB1 in a yet unclear manner. At the level of the AIS, EB1 and EB3 form a complex that interacts with both microtubules and AnkG and possibly acts as a stop signal for the Kv1.2/Kvβ2-containing vesicles. CK2 then leads to release of the vesicle from the microtubules by phosphorylating Kvβ2 which causes its unbinding from EB1. How do the Kv1-containing vesicles recognize their destination site in distal axons and in nodal regions is not known. In addition, the mechanisms by which Kv1 channels get restricted to the juxta-paranodes (JXPN) remains elusive except for an important role of myelin and glial cells. In parallel Kif5B has also been implicated in transporting Kv1.1, Kv1.2 and Kv1.4 to the axon in a mechanism involving the T1 domain, possibly reflecting a similar cargo adaptor role of Kvβ2 interacting with Kv1.2 in this process.

Axonal Kv channels perform several functions such as restricting the propagation of excitation, regulating APs threshold, determining the temporal sharpness of APs generation, enhancing Nav channels availability during repetitive firing and control APs width and subsequent neurotransmitter release (Trimmer, 2015). In this review article, we will focus on Kv1, critical regulators of axonal excitability enriched at the level of the AIS and juxta-paranodes (JXPN) of myelinated axons (Figures 1, 4), which have been extensively studied and exhibit axonal targeting mechanisms that differ from Nav channels.

#### The Voltage-Gated Kv1 Channel

##### Distribution of Kv1 channel in the axon

Various distributions of Kv1 α subunits have been reported in the literature and may vary depending on cell types, dynamic regulatory states but also by sample preparation itself, which has been shown to determine the extent of Kv1 labeling (Trimmer, 2015). Kv1.1 and Kv1.2 are prominently found at the distal AIS of multiple neuronal types and at JXPN in myelinated axonal fibers (Rasband et al., 2001; Poliak et al., 2003; Inda et al., 2006; Kole et al., 2007; Van Wart et al., 2007; Ogawa and Rasband, 2008; Ogawa et al., 2010; Duflocq et al., 2011). At the AIS, Kv1.1 and Kv1.2 expression overlaps with those of Nav1.6 channels (Inda et al., 2006; Kole et al., 2007; Van Wart et al., 2007; Lorincz and Nusser, 2008; Duflocq et al., 2011). The picture for Kv1.4 is less clear. They seem to be preferentially localized at presynapses (Trimmer, 2015) even though they have been shown to co-localize with Kv1.1 and Kv1.2 at the level of the AIS (Ogawa and Rasband, 2008) and to be punctually found at JXPN (Rasband et al., 2001). Kv1.4 channels do not show a strong axonal targeting signal and their expression at the level of the AIS and JXPN might depend on its assembly in Kv1.2-subunit-containing heteromers (Manganas and Trimmer, 2000; Jenkins et al., 2011). Kv1.1 and Kv1.2 mediate low-threshold, slow delayed rectifier currents (Higgs and Spain, 2011) whereas Kv1.4 mediates a fast activating and fast inactivating A-type current (Carrasquillo et al., 2012). The differences between Kv1.1/1.2 and Kv1.4 and the apparent higher association of Kv1.1/1.2 with advanced excitability-related characteristics such as Nav1.6-based AIS and saltatory conduction trough nodes of Ranvier can be explained by the fact that Kv1.4 seems to be an evolutionary ancient member of the Kv1 family (Huang et al., 2014). Finally, heteromeric assembly of Kv1 subunits and β subunits determine the surface expression, subcellular distribution (Li et al., 2000; Manganas and Trimmer, 2000; Jenkins et al., 2011) and kinetics properties of Kv1 complexes (Guan et al., 2006). Kv1.1 and Kv1.2 are highly co-localized at the AIS and nodes of Ranvier, suggesting that the functionally relevant Kv1 complexes in these locations are composed of Kv1.1 and Kv1.2 heteromers, possibly also including Kv1.4 subunits (Figures 1, 4).

##### Targeting Kv1 to the axon

Kv1 channels appear much later in the axon during development as compared to Nav-AnkG complexes (Gu et al., 2006) and Kv1 α subunits do not contain an AnkG binding domain, relying therefore on different mechanisms than Nav channels for their proper axonal targeting.

Various mechanisms have been shown to promote ER exit and surface expression of Kv1 channels (Lai and Jan, 2006). Interestingly, Kv1 exhibit subunit-dependent trafficking properties. Kv1.1 is mostly ER-retained, Kv1.4 is strongly surface expressed but lacks a specific axonal targeting signal whereas Kv1.2 is intermediate, equally ER retained and surface expressed but has the highest specificity for the axonal domain and its surface expression is strongly dependent on co-expression of the Kvβ2 subunit (Li et al., 2000; Manganas and Trimmer, 2000; Tiffany et al., 2000; Manganas et al., 2001; Campomanes et al., 2002; Gu et al., 2003; Jenkins et al., 2011). In neurons, functionally expressed Kv1 channels complexes are mostly, if not all, formed of heteromers that are assembled early in the ER (Shi et al., 1996; Manganas and Trimmer, 2000; Manganas et al., 2001). Thus, enrichment of Kv1 at different subcellular localizations of the axon can be the result of Kv1 α subunits heteromeric assembly combining the ER exit signal from Kv1.4, the axonal targeting signal from Kv1.2 and an unclear role of Kv1.1. In addition, Kv1.4 could further enhance surface expression of Kv1 heteromers by molecularly masking the ER retention signals of the other Kv1 subunits following tetramerization (Manganas and Trimmer, 2000).

Even though Kv α subunits can assemble into functional channels, Kv1 channels *in vivo* are composed of transmembrane Kv α subunits and cytosolic Kvβ subunits, associated in a stoichiometric manner with 4 α subunits—4 β subunits (Pongs and Schwarz, 2010). Kv α and β subunits are assembled early during biosynthesis in the ER (Nagaya and Papazian, 1997) by direct interaction of Kvβ subunits with the T1 tetramerization domain of Kv α subunits (Long et al., 2005). Kvβ subunits greatly extend the intracellular domain of Kv1 channels and provide an interface for intracellular protein binding (Pongs and Schwarz, 2010). They have multiple effects on Kv1 α subunits including regulation of intracellular trafficking, inactivation properties and kinetics (Pongs and Schwarz, 2010). Four Kvβ subunits have been identified, Kvβ1, Kvβ2, Kvβ3 and Kvβ4. Kvβ2 is the predominantly expressed isoform in the brain (Pongs and Schwarz, 2010) and seems to preferentially associate to Kv1.2 (Campomanes et al., 2002; Pongs and Schwarz, 2010).

Over the past 20 years, several studies have revealed that Kvβ2 and its interaction with Kv1.2 is a key upstream determinant of axonal targeting of Kv1 channels. As outlined above, Kv1.2 has a stronger axonal targeting signal than Kv1.1 and Kv1.4 (Gu et al., 2003; Jenkins et al., 2011). The axonal targeting of Kv1.2 and Kv1.2-containing Kv1 heteromers depends on the T1 tetramerization domain, but by a mechanism independent from the proper tetramerization of Kv1 α subunits (Gu et al., 2003). Kvβ2 knockout mice exhibit axon excitability-related impairments such as seizures and tremors (Connor et al., 2005). Kvβ2 subunits are expressed before Kv1 α subunits during development (Gu et al., 2003, 2006; Vacher et al., 2011) and Kvβ2 is required for the proper axonal targeting of Kv1.2 and Kv1.2-containing heteromers (Campomanes et al., 2002; Gu et al., 2006). Kvβ2 acts as a cargo adaptor, or a binding partner for a yet unidentified cargo adaptor, to link Kv1.2 and Kv1.2-containing heteromers with KiF3/kinesin II (Gu et al., 2006). Kvβ2/KiF3 mediated axonal targeting of Kv1.2 was dependent on the plus end tracking protein EB1. Indeed, EB1 and Kvβ2 were shown to be associated and to move together along the axon in live-imaging (Gu et al., 2006). The exact role of EB1 was not directly assessed but it could reflect the fact that Kvβ2-Kv1.2 cargos are tethered on short microtubules polymers by EB1, which are subsequently transported by KiF3 moving along stable, long microtubules (Dent and Baas, 2014). Another possibility is that EB1 is important to signal to the KiF3-Kv1 complex that they reached destination. Indeed, it was shown that EB1 and EB3 interact with AnkG and stabilize both the AIS and microtubules, suggesting that they participate in establishing stable microtubules routes leading to the AIS (Leterrier et al., 2011). Consequently, AIS-enriched kinases such as CK2 would lead to detachment of the cargo vesicle from EB1 and the microtubules (Vacher et al., 2011) prior to membrane insertion. Note that this mechanism can explain the enrichment of Kv1 at the AIS, where Kv1.1, 1.2 and 1.4 colocalize with Nav channels, but at first sight it cannot apply in the nodal region where AnkG-Nav complexes and Kv1 are segregated in different regions, the node and the JXPN respectively. In parallel, KiF5B/kinesin I has been implicated in mediating the axonal transport of Kv1.1, 1.2 and 1.4 (Rivera et al., 2007; Figure 4).

Finally, the complete mechanisms leading to release of Kv1-containing cargoes at specific locations along the axon, particularly in the nodal regions, and their tethering and stabilization at the plasma membrane are not yet understood (Figure 4). Conflicting results have been reported in the literature and the mechanisms might vary between the AIS and the distal myelinated parts, and also between the CNS and the PNS and furthermore among cell types (Buffington and Rasband, 2011; Vacher and Trimmer, 2012; Buttermore et al., 2013; Chang and Rasband, 2013; Trimmer, 2015; Jones and Svitkina, 2016). In addition, the deciphering of nodal Kv1 targeting mechanisms is hampered by the difficulty to properly stain its various molecular partners, possibly reflecting the intense crowding of interacting molecules in these areas (Buffington and Rasband, 2011; Trimmer, 2015). However, the restriction of Kv1 expression at the level of the JXPN appears to critically depend upon myelination and on the integrity of the boundaries formed by the paranodal domain and glia-neuron septate junctions (Boyle et al., 2001; Ishibashi et al., 2002; Poliak et al., 2003; Traka et al., 2003).

#### The Voltage-Gated Kv2 Channel

Two isoforms of Kv2 channels exist, Kv2.1 and Kv2.2 (Trimmer, 2015). Kv2.1 has been recently studied at the level of the AIS of various neurons, including hippocampal principal cells and interneurons, where they form clusters devoid of AnkG staining and segregated from Nav1.6 and Kv1.2 (Johnston et al., 2008; Sarmiere et al., 2008; King et al., 2014; Kirizs et al., 2014). Kv2.1 clusters are distributed relatively uniformly along the AIS but show a different phosphorylation profile in the somatodendritic and proximal AIS as compared with the distal, Nav1.6- and Kv1-enriched, AIS (King et al., 2014) suggesting compartmentalization of their function between these two domains. They associate with cisternal organelles in close vicinity of GABAergic synapses, suggesting that they are implicated in mediating perisomatic inhibition induced changes in axonal excitability (King et al., 2014). In terms of number, Kv2.1 is predominant in the somatodendritic domain (see above). Kv2.2 has been reported to be concentrated at the level of the AIS of MNTB neurons but in a non-clustered distribution, and to support the high frequency firing typical of these neurons (Johnston et al., 2008). Kv2.2 channels have so far not been extensively studied.

##### Changes in functional distribution of axonal Nav and Kv1 channels in physiological contexts

Mechanisms determining the precise patterning of Nav and Kv1 channels at excitable domains of the axon such as the AIS and nodal regions are tightly intermingled with those defining the organization of these structures. Thus, changes in Nav and Kv1 functional distributions in the axon are mostly related to the reorganization of the AIS and nodes of Ranvier (Normand and Rasband, 2015; Yamada and Kuba, 2016).

The plasticity of axonal excitable domains is a relatively recently appreciated phenomenon (Ford et al., 2015; Yamada and Kuba, 2016). The AIS is indeed a highly dynamic structure and both its length (and the corresponding number of Nav and Kv channels) and its relative position to the soma can be modulated in an activity-dependent manner (Grubb et al., 2011; Yamada and Kuba, 2016). These changes strongly affect the excitability of neurons (Yamada and Kuba, 2016). This plasticity of the AIS allows neurons to finely adjust their firing according to their excitatory and inhibitory inputs in physiological but also in pathological conditions (Grubb et al., 2011; Yamada and Kuba, 2016).

Also the composition and functional state of VGCs within the AIS can be subject to modulation. In the avian cochlear nucleus, an enlargement of the AIS following experimental deprivation of auditory inputs is accompanied by a decrease in Kv1 channels, further increasing neuronal excitability (Kuba et al., 2015). Moreover, chronic depolarization of dentate gyrus granule cells induces both AIS shortening and dephosphorylation of Nav channels, with opposite effects on cells excitability (Evans et al., 2015). Recent evidence suggests that nodes of Ranvier are similarly subject to activity dependent changes of their structural and molecular properties (Ford et al., 2015).

Thus, contrary to what was assumed for a long time, excitable domains of axons and the polarized functional distribution of VGCs in these subcellular compartments are highly plastic and further support the elaborated electrogenic properties of neurons and the robust homeostatic balancing abilities of brain networks. The mechanisms involved in these phenomena are only starting to be explored and are likely a result of a complex cross-talk between genetic programs, cell to cell adhesion signals and activity-dependent calcium pathways (Grubb et al., 2011; Susuki and Kuba, 2016; Yamada and Kuba, 2016).

##### Alterations in Nav and Kv channels polarity in pathological conditions

As mentioned above, trafficking and targeting of VGCs are crucial phenomena ensuring a correct processing and integration of electrical signals. Functional alterations or mislocalization of ion channels might thus deeply change the computational properties of a neuron explaining why ion channels deregulations have been observed in several pathologies.

A significant number of studies associate Kv4.2 channels with epilepsy (Bernard et al., 2004; Lugo et al., 2008; Monaghan et al., 2008). Kv4.2-mediated A-type current in CA1 pyramidal cells is reduced after brain injury, most likely explaining the neuronal hyperexcitability observed in epileptic brain (Bernard et al., 2004). In a pilocarpine-induced model of temporal lobe epilepsy the expression pattern of Kv4.2 as well as KChIP2 was altered in response to seizure. The relatively uniform distribution along dendrites of dentate granule cells was shifted to the distal part of the dendrite, whereas the expression of both proteins was deeply reduced along the CA1 pyramidal cells (Monaghan et al., 2008). However, mechanisms underlying the redistribution of the channel along dendrites in pathology are not yet known. The dynamic changes of Kv4.2 channel and its accessory subunits after seizure seem to be a complex phenomenon. In another study from Su et al. (2008) lithium-pilocarpine induced seizures first upregulated the expression of Kv4.2 and KChIP1 along the dendrites of CA1 pyramidal cells, whereas the chronic period was associated with a downregulation of the Kv complex. Traumatic brain injury, a factor associated with the development of epilepsy, also has been shown to diminish Kv4.2 channel expression as well as the Ia current in CA1 pyramidal cells for several weeks after injury (Lei et al., 2012). Determining precisely if and how trafficking or internalization of the Kv4.2 channels at the surface membrane is affected in epilepsy could provide further hints for the understanding of the establishment of the proximodistal gradient.

Ion channel auxiliary subunits seem to be critical for the development of pathologies. Indeed, the Kv2.1 channel auxiliary subunit AMIGO has been shown to be an integral part of the Kv2.1 channel complex (Peltola et al., 2011), and knockout of the AMIGO-encoding gene leads to reduced whole-brain expression of the Kv2.1 channel (Peltola et al., 2016). AMIGO knockout animals display electrophysiological and behavioral features similar to those observed in schizophrenia (Peltola et al., 2016). Interestingly, recent work also points to the involvement of the Kv2.1 channel in the hyperexcitability observed in a mouse model of Alzheimer’s disease (Frazzini et al., 2016). In this model, activation of glutamate receptors leads to the overproduction of Reactive Oxygen Species (ROS) promoting clusterization of Kv2.1 channels in hippocampal neurons. The increase of non-conducting, clustered, Kv2.1 channels makes cells more excitable by changing their firing behavior normally controlled by single Kv2.1 channels (Frazzini et al., 2016).

Within the past decade, the auxiliary subunit DPP6, also called DPPX, has been associated with encephalitis (Boronat et al., 2013; Piepgras et al., 2015). A report from Boronat et al. (2013) has found that patients with encephalitis accompanied by seizure display an autoimmune disorder characterized by the production of anti-DPPX antibodies (Boronat et al., 2013). The altered expression of the auxiliary subunit DPPX caused by the autoimmune reaction could explain the development of seizures on these patients suffering from encephalitis.

Even though alteration of surface localization of ion channels has been mainly associated with pathophysiological conditions, it also has been reported that Kv2.1 channel modulation might underlie neuroprotective mechanisms during ischemia (Misonou et al., 2004, 2005a, 2006, 2008). Kainate-induced seizures induce the redistribution of Kv2.1 channels from clusters to a diffuse single-channel localization along the plasma membrane of pyramidal neurons (Misonou et al., 2004). This declusterization occurs upon calcium influx-mediated activation of calcineurin that dephosphorylates two serine residues (S563 and S603; Figure 2C) of Kv2.1 channels (Misonou et al., 2004). During brain hypoxia/ischemia, calcineurin-dependent dephosphorylation of non-conductive Kv2.1 channel (Misonou et al., 2005a) releases them from clusters, providing a quick and adaptive mechanism allowing neuron to suppress hyperexcitability caused by brain injury (Misonou et al., 2005a), a process that appears to be dependent on neuroglia interaction (Misonou et al., 2008). This potential neuroprotective effect of Kv2.1 channel declustering in the regulation of membrane excitability has been shown to be bidirectional, as suppression of neuronal activity potentiate Kv2.1 phosphorylation and cluster formation (Misonou et al., 2006). Activity-dependent regulation of Kv2.1 channel organization at the plasma membrane level is an excellent example of how ion channel localization might be a crucial component determining the computational properties of a neuron, and how dynamic regulation of channels at the surface is an key mechanism that allows the cell to adapt to its environment.

In pathological contexts, altering the proper distribution and function of Nav and Kv channels in axons has multiple effects on axonal excitability and nervous system function. It is generally difficult to segregate the functional impact of alterations of VGCs localized at the level of nodal regions from those at the level of the AIS because of redundancy in the targeting mechanisms of these channels in these subcellular compartments, as noted previously. In other words, impairment of proper functional expression of VGCs at AIS are often paralleled by defects of the channels at nodes of Ranvier, and reciprocally (Arancibia-Carcamo and Attwell, 2014).

Typically, disrupting Nav channels enrichment and clustering at the AIS and nodes leads to hypoexcitability responsible for various cognitive disorders such as schizophrenia, depression, bipolar disorders and autism (Buffington and Rasband, 2011; Arancibia-Carcamo and Attwell, 2014; Normand and Rasband, 2015; Griggs et al., 2017). In addition, and correlating with the predominant localization of Nav1.1 in the AIS of GABAergic neurons (Ogiwara et al., 2007; Lorincz and Nusser, 2008; Catterall et al., 2010; Tian et al., 2014), the loss of proper localization and function of Nav1.1 is considered to be a factor for the onset of epilepsy caused by a disruption of the inhibitory balance in the brain (Catterall et al., 2010).

The impact of abnormal Kv distribution, particularly Kv1 channels, can lead to failure of axonal conduction because of their stabilizing effects on membrane potential and their effects on Nav channels availability (Trimmer, 2015). In parallel, it is often associated with hyperexcitability syndroms such as epilepsy and ataxia (Buffington and Rasband, 2011; Arancibia-Carcamo and Attwell, 2014; Trimmer, 2015).

Disruption of AIS and nodal structures and proper Nav and Kv localization along the axon is also a hallmark of neurodegenerative diseases such as multiple sclerosis, and axonal injury (Smith, 2007; Buffington and Rasband, 2011; Arancibia-Carcamo and Attwell, 2014; Normand and Rasband, 2015; Griggs et al., 2017). At the nodes of Ranvier, loss of Nav1.2 and Nav1.6 clustering are hallmarks of axonal degeneration (Smith, 2007). In addition, Nav1.2 and Nav1.6 spread expression and up-regulated activity can lead to further axonal damage. Indeed, the increased entry of sodium in the challenged axon can reverse the activity of Na+/Ca2+ exchangers, which will cause toxic accumulation of calcium in the cytoplasm (Smith, 2007).

Multiple pathological mechanisms can lead to impairment of normal AIS and nodes organization and the functional distribution of Nav and Kv channels along the axon (Buffington and Rasband, 2011; Normand and Rasband, 2015; Clark et al., 2016; Griggs et al., 2017). Mutations affecting the cytoskeletal organization typical of these structures, notably AnkG are implicated in a broad range of cognitive disorders (Normand and Rasband, 2015). Altered functions of proteins mediating cell to cell adhesion signaling at the level of the AIS and nodal regions such as Caspr2, but also deficits in myelination in general, are other important pathological factors (Buffington and Rasband, 2011; Arancibia-Carcamo and Attwell, 2014; Griggs et al., 2017). Also auto-immune inflammatory responses can lead to proteolysis of Navs, Kvs and AIS and node components (Griggs et al., 2017).

Finally, in parallel to the identification of β subunits as important regulators of Nav channels targeting and conductance, β subunits are increasingly implicated in Nav channelopathies and emerge as novel pharmacological targets (O’Malley and Isom, 2015). The role of Kv1 β subunits in diseases is less studied than for Nav channels but loss-of-function mutations of Kv1 β2 has been described as an important risk factor for epilepsy (Villa and Combi, 2016).

## Concluding Remarks

Proper expression, segregated distribution and the functional state of Nav and Kv channel isoforms at axonal and somatodendritic domains, and within its subcellular compartments, are critical determinants of the electrogenic properties of a neuron as illustrated by the multiple pathologies associated with Nav and Kv channels (Smith, 2007; Mantegazza et al., 2010; Catterall, 2012; Savio-Galimberti et al., 2012; Shah and Aizenman, 2014; O’Malley and Isom, 2015; Kruger and Isom, 2016; Villa and Combi, 2016). The contribution of their multiple isoforms within single neurons but also among cell types remains to be elucidated (Vacher et al., 2008; Narayanan and Johnston, 2012; Nusser, 2012; Trimmer, 2015). It will also be interesting to determine how the Nav and Kv channel isoforms expression and functional distribution are selected by cell fate and environment and if common rules exist between channel expression, firing properties and neuronal function (Nusser, 2012).

As illustrated in this review article, the mechanisms leading to the multi-layered polarized organization of neuronal VGCs are complex and comprise regulation of biosynthesis, intracellular trafficking, surface expression and biophysical properties. The trafficking pathways mediating the transport of newly synthesized Nav and Kv channels to their specific neuronal compartments are only starting to be unraveled (Gasser et al., 2012; Su et al., 2013; Barry et al., 2014; Akin et al., 2015). In addition, the signaling pathways mediating the detachment of the cargo vesicles from the cytoskeletal tracks and subsequent membrane insertion at specific locations are mostly unknown (Jones and Svitkina, 2016). However, the principles underlying the distribution and targeting of Nav and Kv channels might help us to unravel the mechanisms of polarized distribution of the many other ion channels present in axons and dendrites.

## Author Contributions

All authors contributed to the conception, drafting and critical revision of the article.

## Conflict of Interest Statement

The authors declare that the research was conducted in the absence of any commercial or financial relationships that could be construed as a potential conflict of interest.
